# *Listeria monocytogenes* Dampens the DNA Damage Response

**DOI:** 10.1371/journal.ppat.1004470

**Published:** 2014-10-23

**Authors:** Ascel Samba-Louaka, Jorge M. Pereira, Marie-Anne Nahori, Veronique Villiers, Ludovic Deriano, Mélanie A. Hamon, Pascale Cossart

**Affiliations:** 1 Institut Pasteur, Unité des interactions Bactéries-Cellules, Paris, France; 2 The French National Institute of Health and Medical Research (Inserm), Paris, France; 3 The French National Institute for Agricultural Research (INRA), Paris, France; 4 Institut Pasteur, Laboratoire développement Lymphocytaire et Oncogénèse, Paris, France; 5 Centre National de la Recherche Scientifique (CNRS) Paris, France; Duke University, United States of America

## Abstract

The DNA damage response (DDR) is an essential signaling pathway that detects DNA lesions, which constantly occur upon either endogenous or exogenous assaults, and maintains genetic integrity. An infection by an invading pathogen is one such assault, but how bacteria impact the cellular DDR is poorly documented. Here, we report that infection with *Listeria monocytogenes* induces host DNA breaks. Strikingly, the signature response to these breaks is only moderately activated. We uncover the role of the listerial toxin listeriolysin O (LLO) in blocking the signaling response to DNA breaks through degradation of the sensor Mre11. Knocking out or inactivating proteins involved in the DDR promotes bacterial replication showing the importance of this mechanism for the control of infection. Together, our data highlight that bacterial dampening of the DDR is critical for a successful listerial infection.

## Author Summary

In eukaryotic cells both normal metabolic activities and environmental factors such as UV radiation can cause DNA lesions or mutations. The ability of a cell to restore integrity to its genome is vital, and depends on a signaling cascade called the DNA damage response (DDR) that both senses and responds to the assaults. Bacterial infection is one such assault, but its effect on the DDR of the invaded cell remains elusive. Here we used the bacterial pathogen *Listeria monocytogenes* to study its effect on host DNA damage and its impact on the DDR. Our results show that although *Listeria* is able to induce DNA damage, the ensuing response is surprisingly low, demonstrating that this bacterium is able to dampen the DDR. We have also shown that the listerial toxin listeriolysin O (LLO) is responsible for the observed block in the DDR. In fact, we find that LLO induces protein degradation of the main DNA damage sensor, Mre11, thereby blocking downstream signaling. Furthermore, we have studied the impact of mutating the DDR on the infectious process and find that it negatively regulates infection with *Listeria*. In conclusion, our findings reveal that dampening of the DDR is crucial for a productive infection.

## Introduction

In human cells, the genomic integrity is continuously challenged by environmental and endogenous factors, which induce DNA damage. Cells have evolved an impressive array of repair and signaling pathways to restore the structure of DNA which are collectively termed the DNA damage response (DDR). Briefly, eukaryotic double strand DNA breaks are detected by PARP1 and PARP2 proteins that assemble poly (ADP-ribose) chains on histone H1 and H2B to mediate recruitment of the MRE11-RAD50-NBS1 (MRN) complex, which is assembled at the damaged site and is crucial for engaging the downstream response [1], [2]. The MRN complex recruits the Ataxia-telangiectasia mutated (ATM) protein that contributes to the phosphorylation of H2AX on serine 139 (γH2AX) and of the Mediator of DNA-Damage Checkpoint 1 (MDC1). MDC1 bound to γH2AX acts as an interaction platform for other DDR components including the ubiquitin E3 ligase Ring finger protein 8 (RNF8), which ubiquitinates H2A-type histones and associates with the ubiquitin E2 enzyme UBC13 [3]. H2A ubiquitination is essential for recruitment of another E3 ubiquitin ligase RNF168, itself important for p53 binding protein (53BP1) and breast cancer 1 early onset (BRCA1) foci formation [4], [5]. When the DNA lesion is not repaired, the DDR slows down the cell cycle, leading to either cell cycle arrest, senescence or death [1], [6]. Thus, maintenance of genome integrity is critical for cell homeostasis and defects in the DDR lead to severe genetic diseases or premature ageing [7].

Due to its important role in genomic stability, the DDR is a common target of many viruses [8]. Indeed, the DDR benefits the replication of some viruses, in particular a number of DNA viruses, but for other, such as Herpes virus, the DDR is an obstacle to overcome during infection. Some bacterial pathogens have also been shown to induce genomic instability upon infection, but the impact of the DNA-damage response on infection efficiency is largely unknown [9], [10], [11], [12], [13], [14]. It has, however, been hypothesized that the cell cycle arrest provoked by bacteria-induced DNA damage could prolong bacterial colonization *in vivo* by slowing down the epithelium renewal or exfoliation [14], [15]. More studies are thus needed to understand the action of the DDR components on either bacterial clearance or persistence.

*L. monocytogenes* is the etiological agent of listeriosis, a food borne disease acquired by ingestion of contaminated food. Listeriosis manifests itself as febrile gastroenteritis, or in severe cases as meningoencephalitis, abortion and septicaemia leading to death in 30% of cases [16]. At the cellular level, *L. monocytogenes* is a facultative intracellular bacterium that is well equipped to survive in the cytoplasm of the infected cell. Indeed, *L. monocytogenes* promotes its internalization into host cells via two bacterial proteins InlA and InlB that bind the host receptors E-cadherin and c-Met respectively. Activation of these receptors leads to a series of events involving the recruitment of endocytic effectors, and actin cytoskeletal rearrangements, that induce entry of *Listeria* into the host cell [17]. Once inside the cell, *L. monocytogenes* escapes from the endocytic vacuole using the pore-forming toxin listeriolysin O (LLO), and multiplies in the cytoplasm. There, *Listeria* overexpresses ActA, which allows escape from autophagy and also polymerizes host actin, in order to move and spread to the neighboring cells [18]. Importantly, in addition to mediating vacuole escape, LLO also targets several host functions such as, host gene transcription, mitochondrial dynamics, host protein synthesis or SUMOylation [19], [20]. Thus, *L. monocytogenes* has evolved many mechanisms to manipulate the host to its advantage.

In this study we investigate the effects of *L. monocytogenes* infection on the host DNA integrity. We show that infection with *L. monocytogenes* induces DNA breaks and activates the DDR in host cells *in vitro* and *in vivo*. Strikingly, *L. monocytogenes* is able to dampen activation of the DNA-damage signaling pathway through the bacterial factor LLO. We show that LLO induces the degradation of Mre11, one of the major sensors of DNA damage through a mechanism independent of proteasome degradation. In addition, our results reveal that dampening of the DDR is important for infection as inhibition of proteins implicated in this pathway increases bacterial infection. Thus, our study uncovers a subversion of the DDR during infection by *L. monocytogenes* and a role for the DDR in blocking bacterial proliferation.

## Results

### *L. monocytogenes* induces DNA breaks during infection

**Figure 1:**
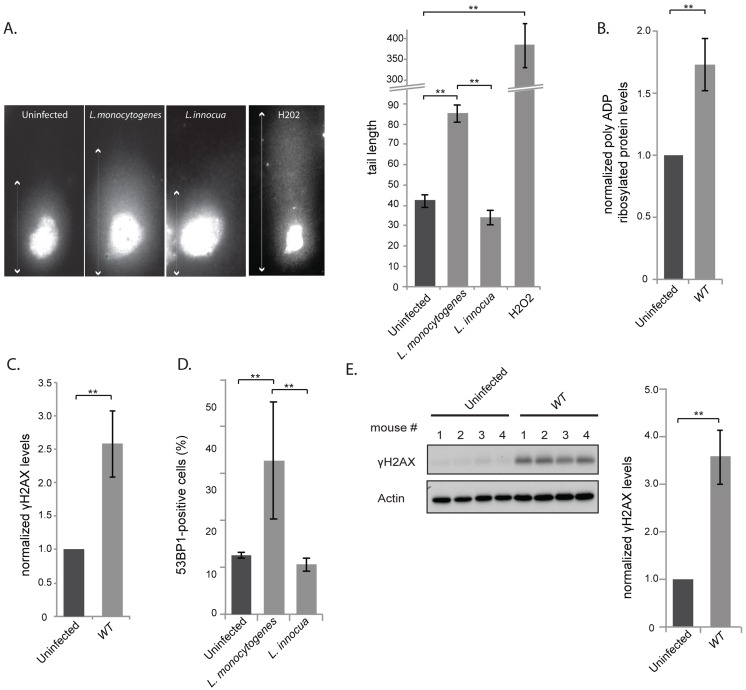
*L. monocytogenes* induces DNA breaks and mildly activates the DNA damage response. (A) Images on the left show comet assays of HeLa cell infected with the indicated strain or treated with purified listeriolysin O or hydrogen peroxide (0.5 mM), and on the right is a quantification of the images. Each box on the left measures 48,3×90 µm, except for the H2O2 treated condition, where the size is double. The white arrows indicate the measured tail length which is recorded and averaged in the histogram on the right. Each bar in the histogram is an average of at least 30 nuclei from at least 3 independent experiments. (B) and (C) HeLa cells were infected with *L. monocytogenes* EGD for 24 h. Cell extracts were collected for immunoblot analysis. The polyADP and γH2AX levels are normalized to actin and to the uninfected condition (n≥3). (D) HeLa cells were infected with *Listeria* for 24 h. 53BP1 foci were visualized by immunofluorescence and quantitated over at least 3 experiments, for a total of more than 500 cells counted. (E) Immunoblot images are shown on the left and quantifications on the right. The left image is shown for 1 experiment on spleen homogenates from C57Bl/6J mice infected with *L. monocytogenes* EGD for 72 h. The histogram on the right integrates 2 experiments (*n* = 8 animals per condition). All quantifications in graphs show the mean +/− SEM (** indicates p<0.01).

To determine whether infection with *L. monocytogenes* induces DNA damage to the host cell, we performed a “comet assay”, a single cell gel electrophoresis assay that is used to show the occurrence of DNA breaks [21]. In this assay cleaved DNA fragments migrate out of the nucleus under the influence of an electric field, whereas most of the DNA does not, or less. By evaluating the DNA comet tail shape and length (arrows on images in figure 1A), one can assess the amount of DNA damage, which can be quantified and averaged over many cell nuclei. Images of control cells that have undamaged DNA and cells treated with H2O2 to induce DNA breaks are shown in figure 1A. Upon infection for 24 h with *L. monocytogenes* the length of the DNA tail was significantly longer than that measured in uninfected cells, or cells incubated with the non-invasive and non-pathogenic *Listeria innocua* (figures 1A). Therefore pathogenic *Listeria* induces DNA breaks during infection.

DNA breaks are detected by the cell, which then initiates the DDR pathway. To determine whether infection with *L. monocytogenes* activates this pathway, we focused on three well-characterized DDR markers: accumulation of poly-ADP ribosylated proteins, phosphorylation of H2AX and increase in the number of 53BP1 foci [22]–[24]. We first studied the levels of poly-ADP ribosylated proteins by western blotting of HeLa cells extracts infected for 24 h with wild type bacteria. A small but significant accumulation of poly-ADP ribosylated proteins could be detected upon infection. Modified proteins ranged from 150 to 200 kDa in size and the accumulation of these proteins was proportional to the multiplicity of infection (MOI) used in the experiment (figure 1B, S1A).

Next, we examined the level of γH2AX, another marker of DNA double stranded breaks. The levels of γH2AX have been shown to increase upon infection with *Shigella flexneri* [9], another invasive bacterium that we took as control. Surprisingly, although *L. monocytogenes* induces DNA breaks upon infection, as shown by the comet assay, the levels of γH2AX showed a significant albeit modest increase in the cells (figure 1C). The increase in γH2AX levels was proportional to the multiplicity of infection used in the experiment (figure S1A), yet lower than that detected upon infection with *S. flexneri* (figure S1B). Interestingly, accumulation of γH2AX was not observed upon infection with the non pathogenic *Listeria innocua*, or with *Staphylococcus aureus* or with *Escherichia coli* K12 (figure S1B).

The last marker we monitored was 53BP1 foci. Resting cells usually show a small number of 53BP1 foci [25]. We therefore counted the number of cells that contained more than 3 foci per cell (figure S2A) and showed that upon infection, there was a higher number of 53BP1 positive cells when infected with *L. monocytogenes* compared to uninfected conditions or to cells incubated with *L. innocua* (figure 1D).

As *Listeria* was inducing DNA breaks and recruitment of associated markers, we investigated whether these events induced an arrest in the cell cycle as generally observed after double strand breaks [26]. We thus synchronized cells with nocodazole and infected them 4 hours after release from the mitotic block. Although a delay of the cell cycle was observed at 16 h and 20 h of infection, this difference was no longer visible at 24 h (figure S2B), suggesting that *Listeria* does not have a significant and long lasting effect on the host cell cycle. Taken together all these results show that although *Listeria* induces a significant level of DNA breaks, as detected by the comet assay, the response to these breaks through the DDR is unexpectedly low.

We further studied whether an increase in γH2AX levels was detectable during an *in vivo* infection. We infected mice for 72 h and harvested the spleens. Figure 1E shows that γH2AX levels displayed a 3.5 fold increase in spleens of infected mice compared to uninfected mice. The larger increase in γH2AX levels observed *in vivo* compared to *in vitro* is probably due to the duration of the infection (72 h vs. 24 h). Therefore we conclude that *L. monocytogenes* induces an increase in γH2AX levels during infection both in tissue-cultured cells and in mice.

**Figure 2:**
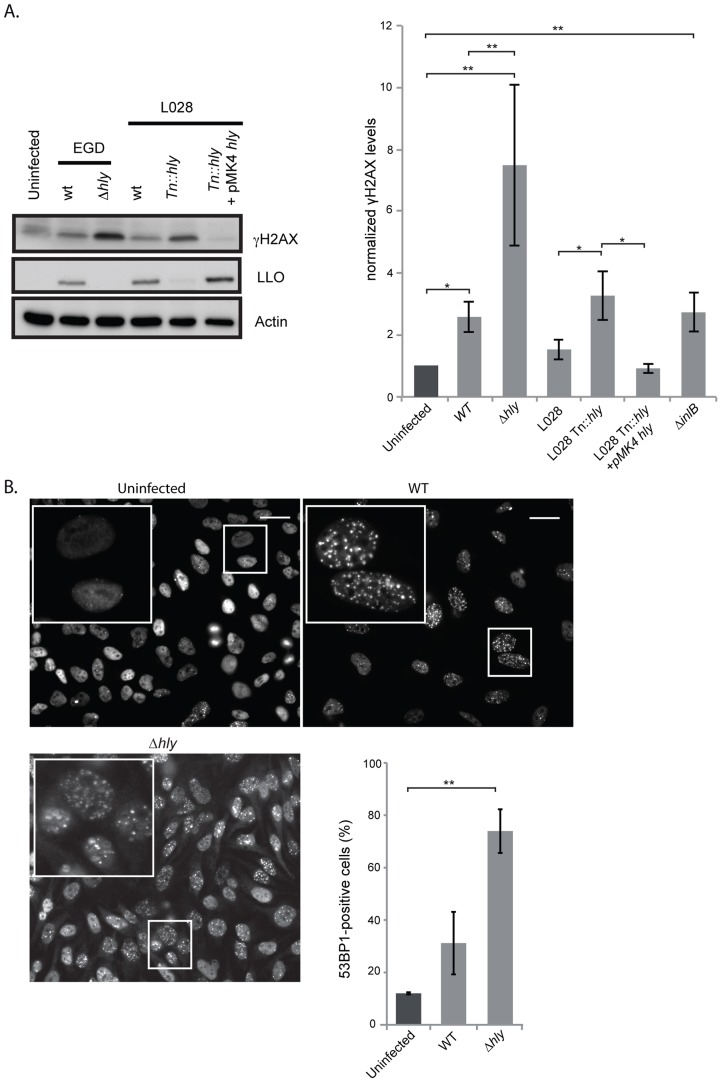
A *Δhly* mutant induces a higher DNA damage response than wild type *Listeria*. (A) HeLa cells were infected for 24 h with the indicated strains of *Listeria*. Cell extracts were collected for immunoblot analysis. A representative immunoblot (left) and a quantification (right) of at least 3 independent experiments are shown. The γH2AX levels are normalized to actin and to the uninfected condition. (B) Immunofluorescence of 53BP1 in HeLa cells infected with *Listeria* for 24 h and quantification of 53BP1-positive cells from at least 3 independent experiments, for a total of more than 500 cells counted. All quantifications in graphs show the mean +/− SEM (* indicates p<0.05, ** p<0.01). Size bars represent 30 µm, insert is 2.5 times larger than box in original image.

To further assess how *L. monocytogenes* induces DNA damage, we investigated using the comet assay, the phenotype of a Δ*inlB* mutant, which lacks InlB the protein crucial for bacterial invasion. Our results show that the size of the comet observed upon infection with a Δ*inlB* mutant was similar to the comet observed upon infection with wild type *Listeria* (figure S3A). Similarly, we treated cells with the actin polymerization inhibitor cytochalasin D to block bacterial entry and measured the size of the cellular comets observed. These results also show that bacterial internalization was not necessary for inducing host cell DNA damage (figure S3A). Consistent with these results, extracellular *Listeria* (either infection with the Δ*inlB* mutant or pretreatment of cells with cytochalasin D prior to infection with wild type bacteria) showed the same level of γH2AX as observed upon infection with *Listeria* able to invade host cells (figure 2 and S3B).

### *L. monocytogenes* deleted for LLO activates the DDR to higher levels than the wild type parent strain

Our finding that extracellular *Listeria* induces DNA breaks during infection led us to investigate the role of the secreted toxin listeriolysin O (LLO), a well-known virulence factor [19]. Using the comet assay, we showed that infection with a Δ*hly* mutant, lacking LLO, led to the formation of the same size comets as when infecting with wild type *Listeria* (figure S3A). Consistent with these results, the purified toxin itself does not provoke DNA breaks (figure S3A). Therefore, the LLO toxin is not important for inducing DNA breaks upon infection with *L. monocytogenes*.

We next studied the effect of LLO on the DDR by measuring the level of H2AX phosphorylation. Surprisingly, as shown in figure 2A, a *Δhly* mutant induced a greater increase in the level of γH2AX than the wild type. These results were reproduced in another cell type and with another strain of *L. monocytogenes*, L028, suggesting that LLO prevents H2AX phosphorylation (figure S4 and 2A). In addition, upon overexpression of LLO in a *Δhly* mutant background, the level of γH2AX was reduced to the same level as that observed in uninfected cells (figure 2A). These data, along with the comet assay results, thus suggested that LLO dampens the DDR downstream of DNA breaks.

We also measured the number of 53BP1 foci upon infection with a *Δhly* mutant. As shown in figure 2B similarly to what is observed for γH2AX, infection with a mutant lacking LLO caused an over 2-fold increase in the number of 53BP1 foci compared to wild type bacteria (around 2000 cells were counted per condition). Together these data show that during infection LLO dampens the DNA damage response, which is normally activated upon DNA breaks.

We further investigated the effect of LLO on the γH2AX levels *in vivo*. For this, we performed peritoneal infections of mice with either the wild type or *Δhly* mutant *L. monocytogenes*. We chose this artificial infection route as it is well known that a *Δhly* mutant is strongly attenuated in the intravenous model of infection and rapidly eliminated [27]. After 6 hours of infection the content of the peritoneum was harvested and the level of γH2AX was assessed by western blot. Upon infection with wild type *Listeria* an increase in the level of γH2AX similar to that occurring in tissue culture was observed, and an even larger increase in the level of γH2AX was detected upon infection with the *Δhly* mutant, even though the same number of bacteria was present in the peritoneal lavage (figure S5). These *in vivo* results therefore confirm our *in vitro* observations, i.e. that LLO prevents phosphorylation of H2AX.

### LLO blocks the DDR induced by cytotoxic agents

**Figure 3:**
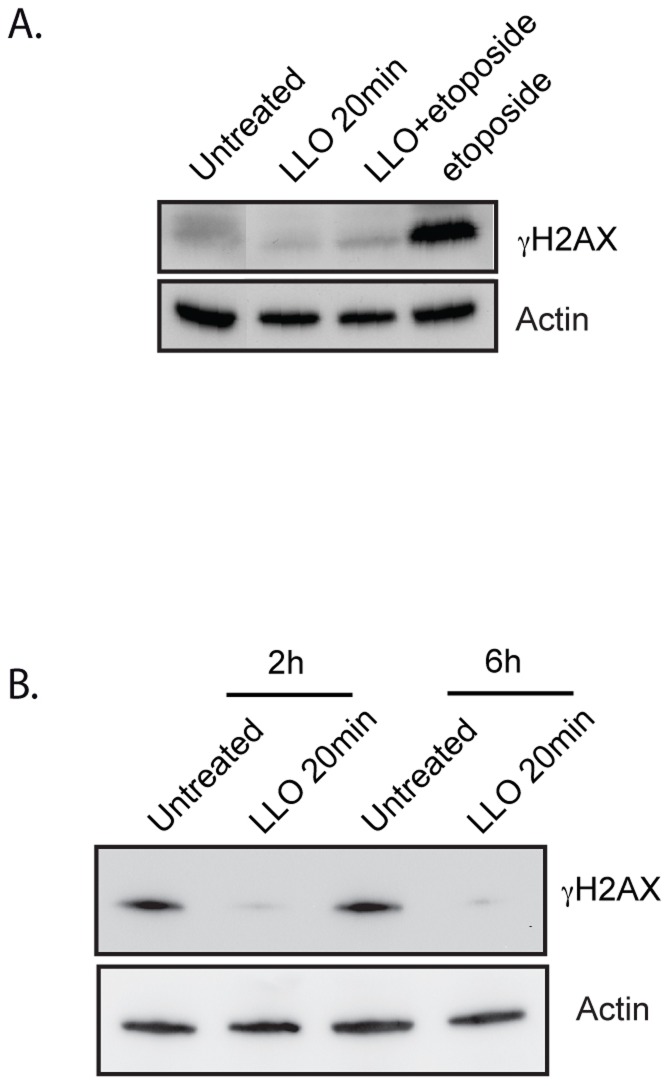
LLO dampens the DDR. (A) and (B) Immunoblots of HeLa cells treated with LLO or left untreated. Toxin treatment was performed for 20 minutes and removed prior to treatment with etoposide (A) or prior to X-irradiation (B). Cells were irradiated with 10 Gray and harvested either 2 h or 6 h after irradiation. Images are representative of experiments performed at least 3 times.

Since our results showed that LLO dampened the DDR induced by infection, we investigated whether LLO could also dampen the DDR induced by known cytotoxic agents such as etoposide or irradiation. To test this hypothesis, we treated cells with purified LLO for 20 min before adding etoposide for 24 h or before X-ray irradiating cells. Our results show that etoposide alone, as expected, induced a large increase in the level of γH2AX, whereas purified LLO on its own did not (figure 3A). Strikingly, upon pre-treatment of cells with LLO, etoposide no longer caused an increase in the level of γH2AX. Similarly, X-ray irradiation, also induces an increase in the level γH2AX within 2 h of the treatment (figure 3B). However, pre-incubation with LLO blocked H2AX phosphorylation induced by X-irradiation (figure 3B). Therefore, the LLO toxin is a potent inhibitor of the DDR.

### LLO induces a proteasome-independent degradation of Mre11

**Figure 4:**
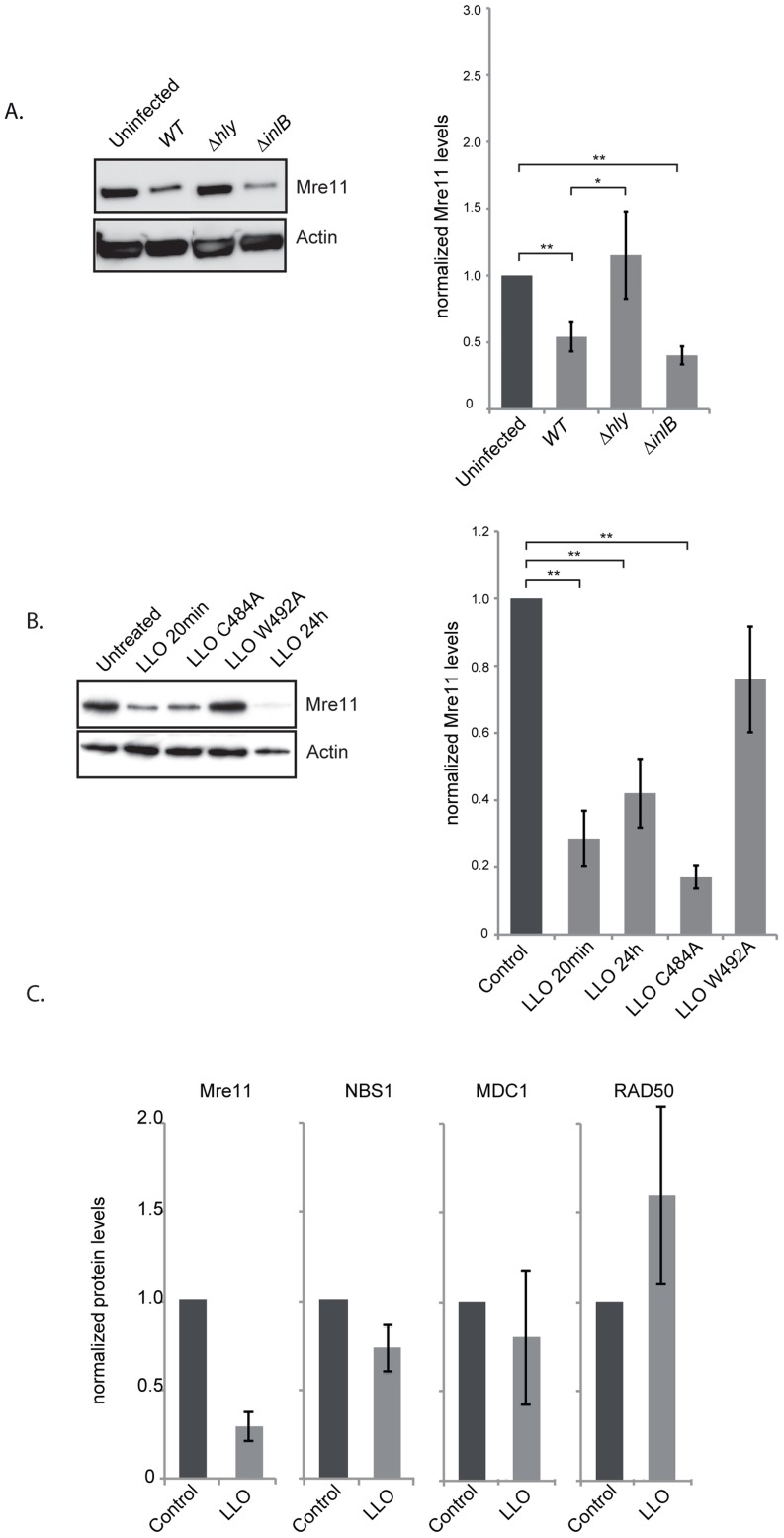
LLO induces a decrease in Mre11 protein levels. (A) Immunoblots (left) and quantification of Mre11 protein levels in HeLa cells infected for 24 h with the indicated listerial strain. (B) Purified LLO (3 nM), or mutant purified LLO (C484A, or W492A) were used for 20 minutes and cells were harvested immediately (LLO 20 min) or after 24 h following removal of LLO (LLO 24 h). Immunoblots (left) and quantification of Mre11 levels (right) are shown. (C) Protein levels of other DDR proteins were assessed by immunoblots in HeLa cells treated with LLO (3 nM) for 20 minutes. All immunoblot quantifications are an average of at least 3 independent experiments, which are normalized to actin and to the uninfected control. All quantifications in graphs show the mean +/− SEM (* indicates that p<0.05, ** p<0.01).

To understand the mechanism by which LLO dampens the DDR we investigated the effect of infection and the effect of the purified LLO toxin on the host proteins, Mre11, RAD50 and NBS1, involved in sensing DNA breaks [6]. Strikingly, after 24 h of infection with wild type *L. monocytogenes* the level of Mre11 significantly decreased (figure 4A). This decrease is dependent on LLO as a *Δhly* mutant does not induce such a decrease (figure 4A). These results can also be observed in JEG3 cells, revealing that the effect is not restricted to HeLa cells (figure S4B). Interestingly, a Δ*inlB* mutant, which is defective in entry into HeLa cells, also induces a decrease in the level of Mre11 similarly to the wild type strain further suggesting that secreted LLO is necessary for this effect (figure 4A). We further investigated whether purified LLO was sufficient to induce Mre11 degradation. As shown in figure 4B incubation of cells with a sub-lytic dose of LLO (3 nM) for 20 minutes was sufficient for decreasing Mre11 levels more than 4 fold. Interestingly, whereas LLO induced a decrease in the levels of Mre11, the levels of RAD50, NBS1, or another DDR protein, MDC1 were unchanged (figure 4C). LLO is a pore-forming toxin. We therefore investigated whether pore formation was necessary for Mre11 degradation by incubating cells with mutant forms of LLO which are affected in their hemolytic activity [28]. A point mutation in LLO at residue 492 (W-A) renders LLO cytolytically inactive, whereas a mutation at residue 484 (C-A) only reduces LLO's activity by 25%. As shown in figure 4B the LLO C484A mutant induces the same decrease in Mre11 levels as wild type LLO, whereas the LLO W492A mutant no longer induces Mre11 degradation. Therefore, pore formation by LLO is required for the impact on Mre11 protein levels. Therefore LLO induces a specific degradation of Mre11, an effect mediated through pore formation at the plasma membrane.

**Figure 5:**
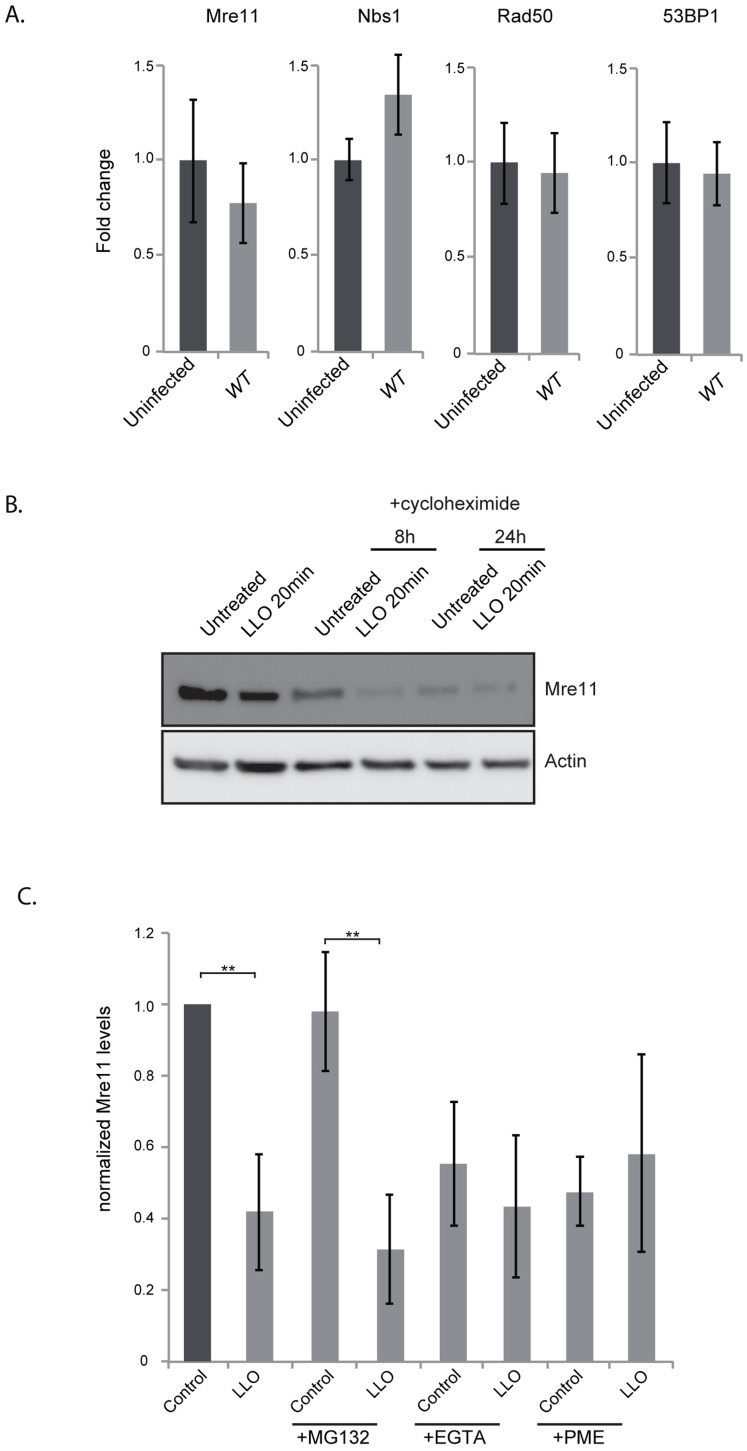
LLO induces degradation of Mre11 in an aspartyl-protease dependent manner. (A) Quantitative PCR experiments comparing uninfected HeLa cells to cells infected for 24 h with wild type *L. monocytogenes*. Error bars are standard deviation as calculated by the Qiagen RT2 Profiler PCR analysis program. (B) Immunoblot of HeLa cells treated with cycloheximide for the indicated times. (C) Quantification of immunoblots of cells treated with the indicated inhibitors. For all experiments LLO was used at 3 nM for 20 minutes. All quantifications in graphs show the mean +/− SEM (** indicates p<0.01).

We next aimed at understanding the mechanism underlying the decrease in the protein level of Mre11. By quantitative PCR we determined that infection was not decreasing transcription levels of the *Mre11* gene, or any other gene of the DNA sensing complex (figure 5A). To determine whether LLO was inducing a block in protein synthesis, we performed experiments using cycloheximide which blocks translational elongation. Our results show that cycloheximide did not prevent the LLO-induced decrease in Mre11 levels strongly suggesting that LLO was triggering a degradation of Mre11 (figure 5B). We further used chemical inhibitors to uncover the mechanism of Mre11 degradation. MG132 was used to block the proteasome, however this inhibitor did not block LLO-mediated Mre11 degradation (figure 5C). However, we found that using a cell permeable inhibitor of aspartyl-proteases, pepstatin-A-methylester (PME), or incubating cells in EGTA, which quenches extracellular calcium, blocked LLO-mediated Mre11 degradation (figure 5C). Therefore, our data show that LLO induces a proteasome-independent degradation of Mre11, a mechanism that appears to require a calcium dependent aspartyl-protease.

### Dampening of the DDR is required for productive infection

**Figure 6:**
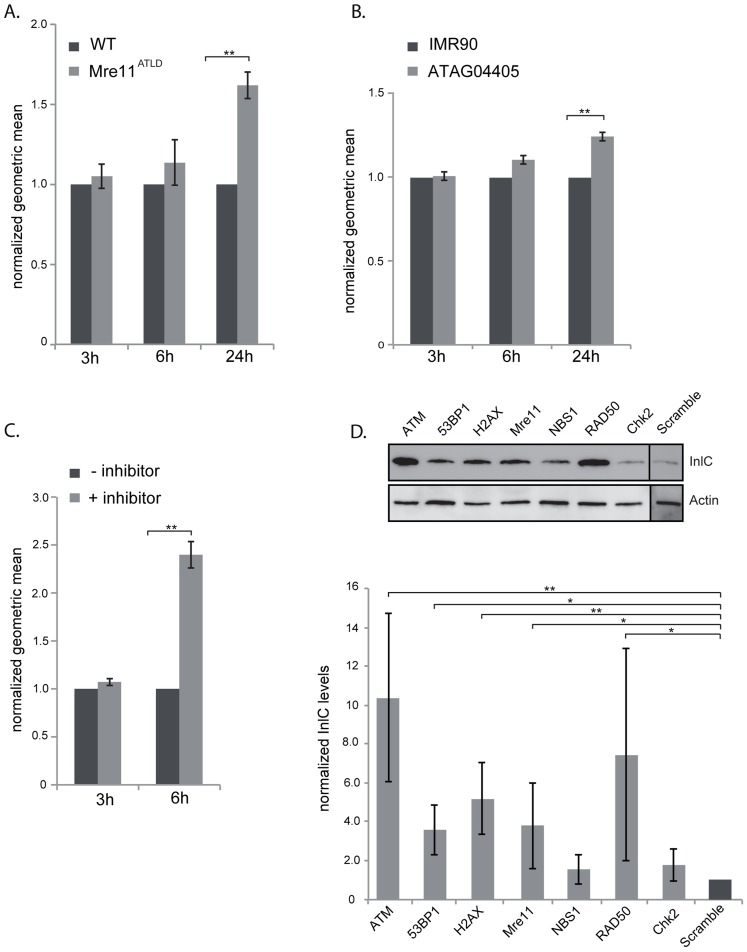
The DNA damage response is negative regulator of infection. (A) WT MEFs or Mre11^ATLD^ MEFs, or (B) WT human fibroblasts (IMR90) and fibroblasts bearing a mutation in ATM (ATAG04405) were infected with *L. monocytogenes* expressing GFP for the indicated times. FACS analysis was performed on infected cells and the geometric mean of 10,000 cells was measured using the FlowJo software. Results are normalized to uninfected cells and averaged over at least 3 independent experiments. (C) HeLa cells were infected with *L. monocytogenes* expressing GFP for the indicated times. The ATM inhibitor was added (+ inhibitor) 1 h after the start of infection. FACS analysis was performed on infected cells and the geometric mean of 10,000 cells was measured using the FlowJo software. Results are normalized to uninfected cells and averaged over at least 3 independent experiments. (D) HeLa cells were transfected with the indicated siRNA and infected with *L. monocytogenes*. Cells were collected after 24 h of infection and immunobloted for the Listerial protein InlC (top). Quantifications (bottom) are normalized to actin and to the scramble sample over at least 3 independent experiments. All quantifications in graphs show the mean +/− SEM (* indicates that p<0.05, ** p<0.01).

Our data show that *L. monocytogenes*, through the activity of LLO, dampens the DDR during infection. To determine whether dampening of the DDR was important for infection, we first focused on *mre11*, whose gene product is degraded during infection. We infected mouse embryonic fibroblasts (MEFs) from Mre11^ATLD^ mice, in which the *mre11* gene is truncated [29], with *L. monocytogenes* expressing GFP and compared, infection of these cells to infection of wild type MEFs by FACS analysis. We monitored the number of bacteria per cell through detection of GFP levels, which are directly proportional to the number of bacteria [30]. At early times of infection, i.e. 3 and 6 hours post infection, there was no difference in the GFP levels detected in wild type MEFs and Mre11^ATLD^ MEFs, indicating that there is no difference in entry and early stages of bacterial replication between the two cell types (figure 6A). However, at 24 hours of infection there was a significantly higher level of GFP detected in Mre11^ATLD^ MEFs compared to wild type MEFs (figure 6A). These results show that an impaired Mre11 function promotes bacterial growth at late infection time points, suggesting that Mre11 dependent DDR controls *L. monocytogenes* proliferation.

Mre11, which is part of the MRN complex, is important for recruiting ATM, a kinase that contributes to the phosphorylation of H2AX [1]. We thus also tested the role of ATM in the infectious process by two different approaches. We first infected wild type human fibroblasts (IMR90) and fibroblasts isolated from ataxia-telangiectasia syndrome patients which have a mutation in the *ATM* gene. Similarly to the results obtained in Mre11^ATLD^ MEFs, we observed at 24 hours of infection a higher GFP signal in *ATM* deleted fibroblasts compared to the wild-type cells (figure 6B). The difference in the GFP signal was only visible at 24 hours of infection and not at 3 or 6 hours (figure 6B). We further tested the role of ATM by infecting cells treated with a pharmacological inhibitor of ATM (KU60019). In order to keep the incubation time with the inhibitor low, we only monitored infection at 3 and 6 hours of infection. Our FACS analysis showed that at 3 hours of infection the ATM inhibitor had no effect on the GFP levels recovered from cells. However, at 6 hours of infection, the mean fluorescence detected in cells treated with the ATM inhibitor was more than twice that of untreated cells (figure 6C). These data therefore confirm the results obtained with human fibroblasts. To further demonstrate the inhibitory role of ATM on infection we performed another assay in which cells are transfected with siRNA against ATM and infected with *L. monocytogenes*. Cells are then recovered and monitored by western blot for the levels of InlC, a listerial protein overexpressed when bacteria are intracellular which provides a direct read out of bacterial proliferation [31]. As shown in figure 6D, the level of InlC is over 3 fold higher in cells knocked down for ATM compared to the control siRNA treated cells. These results further demonstrate the inhibitory role of ATM during a *Listeria* infection.

To extend our analysis to other DDR proteins, we treated HeLa cells with siRNAs against 53BP1, H2AX, Mre11, NBS1, RAD50 and CHK2. As shown in figure 6D, the level of InlC is significantly higher in cells knocked down for 53BP1, H2AX, Mre11, and RAD50 compared to control siRNA treated cells. Intracellular bacteria and InlC can also be visualized by immunofluorescence microscopy. This technique also revealed higher levels of infection in 53BP1, H2AX and ATM knocked down cells (figure S6). Taken together, these data establish that inhibition of the DDR promotes *L. monocytogenes* infection.

## Discussion

**Figure 7:**
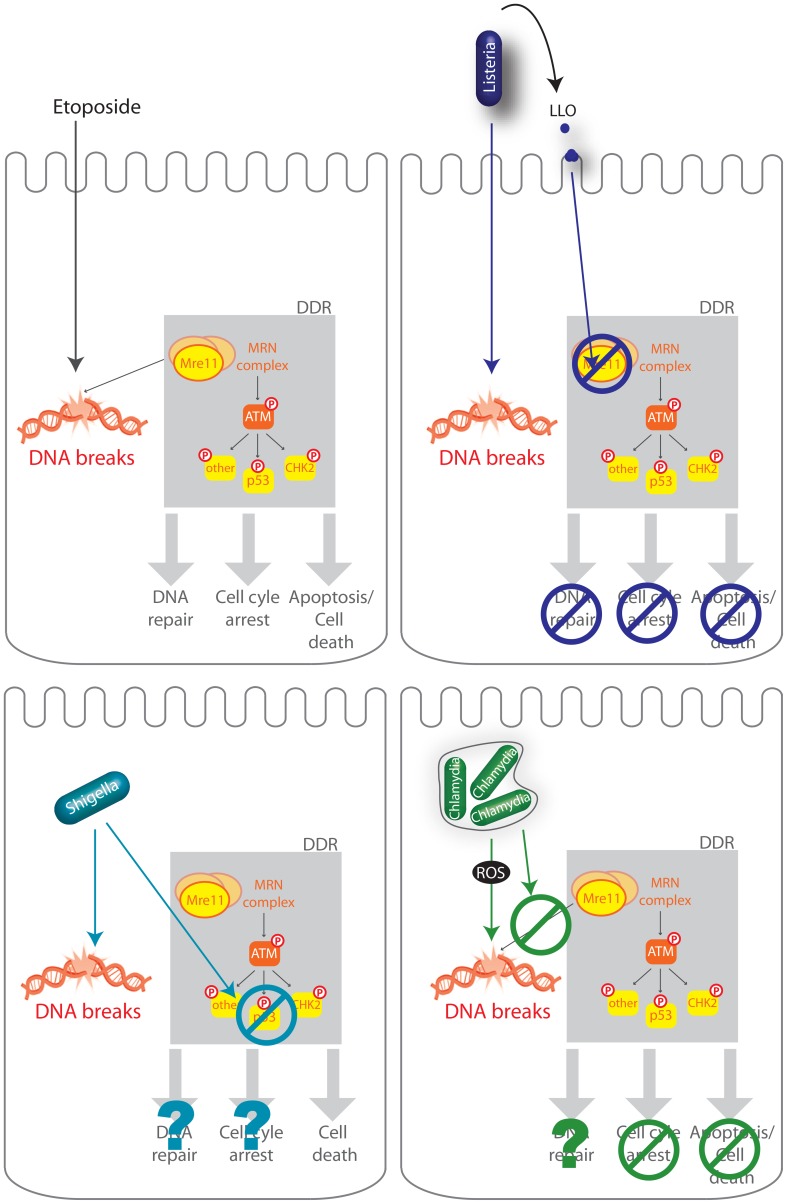
Schematic representation of several infectious processes affecting the DDR. The DDR regulates DNA repair, cell-cycle checkpoints in order to halt cellular proliferation, or, if the damage is too important, cell death. Etoposide is a cytotoxic agent which causes DNA strand breaks. DNA lesions are recognized by the MRN complex (MRE11, RAD50, and NBS1) and leads to the phosphorylation of ATM and further activation of the DDR including phosphorylation of p53, CHK2, and other substrates. *L. monocytogenes* (*Listeria*), *S. flexneri* (*Shigella*), *C. trachomatis* (*Chlamydia*) all induce DNA breaks during infection but impair the DDR using different mechanisms. *L. monocytogenes* LLO, through pore formation induces degradation of Mre11, an associated block in the DDR, no cell cycle arrest, and no cell death (Stavru et al., 2011). *S. flexneri* infection leads to a decrease in the levels of p53 and cell death (Bergonioux et al., 2012), whereas *C. trachomatis* blocks recruitment of the MRN complex and blocks cell cycle arrest (Chumduri et al., 2013).

In this study, we report that the bacterium *L. monocytogenes* provokes DNA breaks in its host while at the same time controlling the ensuing host DDR. Indeed, we found that although wild type bacteria modestly induce the DDR, a *Δhly* mutant lacking LLO did so to a significantly higher level. We further reveal that LLO dampens the DDR, through degradation of the host protein Mre11 (figure 7). Interestingly and consistent with these observations, we find that deletion or inhibition of proteins implicated in the DDR lead to higher levels of infection demonstrating for the first time that the DDR can be a negative regulator of a bacterial infection.

Several bacteria have been reported to induce host DNA damage, a phenotype which had not yet been studied during a *Listeria* infection. Extracellular pathogenic bacteria such as *Pseudomonas aeruginosa*, *Neisseria gonorrhoeae, E. coli*, or *Helicobacter pylori* and intracellular bacteria such as *S. flexneri* and *Chlamydia trichomatis* are able to induce host DNA double-strand breaks (DBS) [9], [10], [12]–[14]. Very recently a report has shown that *Listeria monocytogenes* delays the host cell cycle by increasing the levels of host DNA strand breaks [32]. However, in most cases, the bacterial factor(s) responsible for inducing these DNA breaks has/have not been identified. To our knowledge, only two bacterial virulence factors have been shown to directly induce DNA breaks: Cytolethal distending toxin (CTD) produced by several Gram negative pathogens such as *E. coli*, and Typhoid toxin (TT) that is expressed by the intracellular *S. typhi* [15], [33], [11]. Both types of toxin are related and share a common subunit, CdtB, responsible for DNA breaks. However, no homologues of these virulence factors are present in *L. monocytogenes*. It has recently been reported that *C. trachomatis* could induce DSB through generation of reactive oxygen species (ROS) [10]. We explored this possibility during infection with *L. monocytogenes* using the DCFDA reporter, but failed to observe ROS production under our conditions during infection. Thus, ROS production does not appear to contribute to DNA breaks induced by *Listeria*. A very recent report using multiple bacterial, fungal and plant pathogen species which induce DSBs in plant DNA illustrates the complexity of identifying the cause of pathogen-induced DNA damage [34] and that other factors besides ROS are involved. In our case, we still observe the same increase in γH2AX levels during infection with *L. monocytogenes* deleted for *inlB* and impaired in cell invasion, and therefore this suggests that a secreted virulence factor or a surface component is necessary to induce the observed DNA breaks.

Along with the genotoxic stress induced by *S. flexneri* and *C. trachomatis*, both bacteria were also shown to impair the DDR (figure 4). *Shigella flexneri* infection leads to DNA breaks, γH2AX accumulation along with a degradation of the p53 protein, which blocks the p53-dependent DNA repair response [9]. Similarly, *C. trachomatis* was very recently shown to induce extensive double strand breaks during infection with no associated cell cycle arrest [10]. Indeed, *C. trachomatis* blocked the formation of 53BP1 foci and phosphorylation of Mre11 through an unknown mechanism. Remarkably, in our report we find that *L. monocytogenes*, via LLO, also prevents excessive DNA damage signaling, similarly to *S. flexneri* and *C. trachomatis*, but through a different mechanism. Interestingly, we show in this report that the DDR is a negative regulator of a listerial infection. Although the inhibitory effect of the DDR on infection had been suggested for *S. flexneri* and *C. trachomatis*, this had not been shown experimentally. Our results therefore indicate that dampening of the DDR might be a common and beneficial feature of multiple intracellular pathogen infections.

It is clear that viruses, being obligate intracellular pathogens have an advantage to block the host DDR in order to inhibit cell-cycle checkpoints and p53-mediated apoptosis and keep the cell alive. However, inhibition of the DDR by a bacterial pathogen such as *L. monocytogenes*, which can survive very well without the host cell, is less intuitive. Nevertheless, It is well known that the DDR induces activation of NF-κB [35], [36]. Furthermore, a very recent report shows that DNA damage induces an immune response [37]. In addition, it has been suggested that the Mre11 protein could be a sensor of exogenous DNA leading to activation of type I interferon [38]. Therefore, our data along with a growing body of evidence suggests that the DDR could have an important role in inducing inflammation during infection.

To our knowledge, this is the first report to show that a bacterium induces Mre11 degradation. Mre11 degradation has only been shown to occur during infection with a virus. Indeed, infection with adenovirus has been shown to result in both reorganization and degradation of members of the Mre11–Rad50–NBS1 complex [39]. Furthermore, and similarly to our findings, inhibition of the DDR is essential for adenovirus infection as in the absence of this block, viral replication is inhibited [39]. Therefore, the DDR is a negative regulator of adenovirus infection. Likewise, in the case of *Listeria*, we show that downregulation of Mre11 or ATM or H2AX favors infection. This is the first report showing that the DDR has an impact on bacterial infection.

Finally we show here that LLO induces a decrease in the levels of Mre11 in a pore dependent manner. Indeed, point mutants of LLO which are reduced in their hemolytic activity lead to a lesser effect on Mre11 and γH2AX compared to native LLO. LLO is a pore forming toxin which was originally shown to be essential for *Listeria* escape from the internalization vacuole [40]. Since then, LLO has been shown to have many different extracellular activities on the host cell [19]. Remarkably, the pore formation of LLO has been shown to lead to degradation of at least 2 other proteins during infection, the catalytic subunit of the human telomerase complex (hTERT), and the human E2 SUMO enzyme Ubc9 [20], [41]. Although the mechanism by which hTERT levels were decreased remains to be determined, the degradation of Ubc9 was shown to be partially dependent on an aspartyl-protease. Similarly, we find here that Mre11 is degraded, in a manner that appears to be dependent on an aspartyl-protease. Therefore, the downstream events of LLO pore formation leading to cellular protein degradation are beginning to emerge, however, the specificity of the targeted protein and the molecular mechanism by which degradation occurs remain elusive. Surprisingly, we find that Mre11 levels remain low 24 h after removal of LLO, even though pores have been shown to be repaired very rapidly [42]. The mechanism by which the long lasting effect on Mre11 is achieved will be of particular interest in future studies, and have a wide ranging impact on understanding the long term effects of bacterially induced DNA damage.

## Materials and Methods

### Ethics statement

The mouse strained used was C56BL/6N. All the procedures were performed in accordance with protocols approved by the Animal Experimentation Ethics Committee of the Institut Pasteur (permit #03-49), in application of the guidelines of the European Commission for the handling of laboratory animals, Directive 2010/63/EU (http://ec.europa.eu/environment/chemicals/lab_animals/legislation_en.htm). Protocols were approved by the veterinary staff of the Institut Pasteur animal facility and were performed in compliance with the NIH Animal Welfare Insurance #A5476-01 issued on 31/07/2012.

### Cell lines, bacterial strains

HeLa (CCL-2) and Jeg-3 (HTB-36) cell lines from American Type Culture Collection (ATCC) were grown in Minimum Essential Medium (MEM GlutaMax; Invitrogen) supplemented with 10% Foetal Bovine Serum (FBS) at 37°C in a 10% CO_2_ atmosphere. LoVo (CCL-229) cells from ATCC were cultured in Ham's F12K, 2 mM glutamine (Invitogen) with 10% FBS. MEF and human fibroblasts were grown in Dubelcco's Modified Eagle Medium (DMEM GlutaMax; Invitrogen) supplemented with 10% FBS. Purified LLO was obtained as described previously [43] and was used at 3 nM. When needed cells were treated with etoposide (Sigma) at 40 µM or hydrogen peroxide at 0.5 mM.

*Escherichia coli* K12, *E. coli* K12+invasin from *yersinia pseudotuberculosis* (BUG2940) and *Salmonella typhimurium* sri11 (BUG3044) were grown in Luria-Bertani (LB) medium (BD Difco) at 37°C. *Listerias* strains and *Staphylococcus aureus* (RN6390) were grown in brain heart infusion (BHI) medium (BD Difco) whereas *Shigella flexneri* M90T (BUG2505) were cultured in tryptic soy broth (TSB) medium (BD Difco) at 37°C. The *Listeria* strains used in this study were *L. innocua* (BUG499), *L. monocytogenes* EGD (BUG600), *L. monocytogenes* EGD-GFP (BUG2539), *L. monocytogenes* EGDΔ*hly* (BUG2132), *L. monocytogenes* EGDΔ*inlB* (BUG1047), L. *monocytogenes* L028 (BOF 343), L. *monocytogenes* L028 *Tn*::*hly* (BOF 415), and *L. monocytogenes* L028 *Tn*::*hly* (BOF 415) overexpressing LLO (BUG 210).

### Cell treatments

For synchronization, HeLa cells were blocked in mitosis with 300 nM nocodazole (Sigma) for 22 h. Cells were infected 4 h after release in nocodazole free growth medium. Etoposide treatment of cells was performed for 24 h using a concentration of 40 µM. Cells were exposed to 10 Gray X-ray irradiation using an Xstrahl RS320 irradiator. Cycloheximide treatment was performed using a concentration of 100 µM.

MG132 treatment was performed for 5 h prior to infection and was used at a concentration of 10 µM. Pepstatin-A-methylester (PME) was used for 1 h prior to infection at a concentration of 200 µM. 20 mM EGTA was added to cells upon infection.

Cytochalasin D treatments were performed for 15 minutes prior to infection at a concentration of 5 µg/ml.

### Bacterial infections

For *in vitro* infection: Bacteria were cultured overnight and then sub-cultured 1∶10 in BHI medium for 2 h at 37°C. Bacteria were then washed three times in medium without serum. Prior to infection, HeLa cells were incubated in medium without serum. After addition of bacteria, cells were centrifuged at 1,000× *g* for 1 min and incubated at 37°C for 1 hour. When not specified, infection was achieved with a multiplicity of infection (MOI) of 50. Infected cells were then grown in growth medium supplemented with 20 µg/ml gentamicin.

*In vivo* infections were performed by intra venous injection of 10^5^ bacteria per animal. Experiments were performed according to the Institut Pasteur guidelines for animal experimentation. Spleens were collected and homogenized. CFUs were evaluated from homogenates and one volume of homogenate was diluted into 4 volumes of Laemli buffer sonicated and loaded on SDS–polyacrylamide gels for immunoblotting experiments.

Concerning peritoneum infection, mice were infected with 1.10^7^ CFU for 6 h. The peritoneum content was washed with PBS to harvest bacteria and cells. A part of this content was treated or not with 0.1% triton X-100 and was plated on BHI agar plates to quantify the number of bacteria by counting CFU. Remaining content was centrifuged and cell pellet was lyzed in 2× laemmli buffer.

### Comet assay

HeLa cells were grown adherent to tissue culture plates and infected or treated as detailed in figure. Cells were then treated as recommended in the comet SCGE kit (enzo #ADI-900-166). Briefly, cells were scraped, washed in cold PBS, mixed with LMAgarose and spread on Trevigen comet slides. Agarose embedded cells are then lysed and run on a horizontal electrophoresis apparatus on 32 V for 20 minutes. Slides are then dried and stained with DAPI. Image acquisition of comets was done using a 63× objective on an inverted fluorescence microscope. Image analysis was performed using the comet assay plug-in for Image J.

### Flow cytometry, immunofluorescence and immunoblot

Bacterial infection was assessed by flow cytometry using the BD Cytofix/cytoperm kit (BD Biosciences). Briefly, cells were trypsinized and fixed for 20 minute with fixation and permeabilization solution. Cells were then harvested in PBS for analysis. For cell cycle distribution, cells were scrapped, resuspended in PBS and fixed in 70% ethanol for at least 1 h at −20°C. Cells were washed and re-suspended in PBS containing 15 mg/ml of propidium iodide and 100 mg/ml of RNase A. Flow cytometry acquisitions were performed on a FACScalibur flow cytometer (Becton Dickinson). Data were analysed using FlowJo software.

For measuring infection, *Listeria monocytogenes* expressing GFP were used as described in [31]. Infected cells were trypsinized and analyzed on a FACScalibure. Data was analyzed using the FlowJo software.

To quantify positive cells for 53BP1 or γH2AX by immunofluorescence, infected cells were washed and fixed in 4% paraformaldehyde. After permeabilization, 53BP1 or γH2AX proteins were stained with an anti-53BP1 antibody (Novus biologicals) or anti-γH2AX (#9718, Cell Signaling), and a 546 nM goat anti-rabbit antibody (Invitrogen). References for the antibodies used in our studies are the following: NBS1 (Novus biologicals NB100-143), MDC1 (Novus biologicals NB100-395), RAD50 (Novus biologicals NB100-147).

For Western blot analysis, cells were lysed with 2× Laemmli loading buffer (124 mM Tris-HCl [pH 6.8], 4% SDS, 20% glycerol, 0.02% bromophenol blue, 0.03% dithiothreitol [DTT]), sonicated for 2 s, and then boiled for 5 min. After running on gels, proteins were transferred on a nitrocellulose membrane (GE Healthcare) that was then blocked in 10% milk. The primary antibodies were anti-actin (A5441; Sigma), anti-phospho H3 (ser10) (05-817; Upstate, Lake Placid, NY), anti-Poly (ADP-ribose) (MAB3192 Millipore), anti-γH2AX (#9718, Cell Signaling), Mre11 (#4895, Cell Signaling). Rabbit polyclonal antibodies against InlC and LLO (R176) were raised by immunizing rabbits with purified recombinant LLO and InlC proteins. The sera were then purified against InlC and LLO to obtain the purified antibodies (43). Quantification of immunoblots was performed using a G:Box Syngene and the associated software.

### Quantitative PCR

mRNA was extracted from uninfected cells and cells infected with *L. monocytogenes* using the Qiagen RNeasy kit. cDNA was then synthesized using the Qiagen RT^2^ first strand kit. Real-time PCR was performed using Qiagen RT^2^ PCR arrays (DNA damage array PAHS-029Z) on a Biorad CFX384. Data was analysed using the Qiagen RT^2^ Profiler PCR array data analysis.

### siRNA

HeLa cells were reverse transfected with commercial siRNA for ATM (L-003201, Thermo scientific), CHK2 (L-003256, Thermo scientific), 53BP1 (s14313, Ambion), H2AX (s226270, Ambion). Briefly, 25 nM siRNA is mixed with 4 µl Lipofectamine RNAiMAX (Invitrogen) in a 400 µl volume. After 15 minutes of incubation 5.5×10^4^ cells were added per well of a 6 well plate. Infections were done 48 h after siRNA treatment for an additional 24 h.

### Statistical analyses

When not specified, results are expressed as the means of three independent experiments. The error bars represent the standard errors of the mean (SEM). The analyses were performed with Student's *t* test, and the statistical significance was established at *P* values of <0.05 or <0.001 (indicated by one or two asterisks respectively).

## Supporting Information

Figure S1 **Levels of poly-ADP ribosylated proteins and γH2AX as a function of multiplicity of infection and bacterial strains.** (A) HeLa cells are infected with *L. monocytogenes* using the indicated multiplicity of infection (MOI) and harvested for immunobloting after 24 h of infection. (B) HeLa cells are infected with the indicated bacterial strains and harvested for immunobloting after 24 h of infection.
(PDF)

Figure S2 **Effect of infection with** ***L. monocytogenes*** **on 53BP1 foci and the host cell cycle.** (A) HeLa cells are infected with *L. monocytogenes* for 24 h. Immunofluorescence using an antibody specific to 53BP1 is shown. Arrows show cells with more than 3 foci of 53BP1 which are quantified in figure 1. Scale bar is 30 µm, insert is 2.5 times larger than box in original image. (B) Cell cycle quantification of HeLa cells infected (+) or not (−) with *L. monocytogenes* for the indicated times. In the table the numbers used to draw the graph are shown +/− SEM. (* p<0.05).
(PDF)

Figure S3 ***L. monocytogenes* mediates its effect on the DDR from the outside of the cell.** (A) Quantification of comet assays performed on HeLa cells infected with the indicated strain for 24 h. Cytochalasin D treatment was performed for 15 minutes prior to infection. Each bar in the histogram is an average of at least 30 nuclei from at least 3 independent experiments. Quantifications show the mean +/− SEM (** indicates p<0.01). (B) Immunofluorescence of γH2AX. Each box is 100 µm in length. (C) Immunoblot image representative of 3 independent experiments.
(JPG)

Figure S4 **γH2AX and Mre11 levels in JEG3 cells.** (A) and (B) JEG3 cells were infected with the indicated strain of *Listeria* for 24 h. Cell extracts were harvested for immunoblotting. Quantifications are normalized to actin and to the uninfected sample. All quantifications in graphs show the mean +/− SEM (** p<0.01).
(PDF)

Figure S5 **γH2AX** ***in vivo.*** Immunoblot of peritoneal content from C57Bl/6J mice infected with *Listeria* for 6 hours. *n* = 6 mice per condition. Quantifications in graphs show the mean +/− SEM (* p<0.05, ** p<0.01). Recovered colonie forming units for each conditions were as follows: WT, 46+/−29 and Δhly, 45+/−19.
(PDF)

Figure S6 **Infection levels in siRNA treated cells.** HeLa cells are transfected with siRNA (indicated above each image) and infected with *L. monocytogenes* for 24 h. Immunofluorescence is performed with anti-InlC antibody (green), which measures the level of infection and phalloidin (red), which stains for actin and shows the cell cytoskeleton as well as bacterial induced actin polymerization.
(JPG)
