# Supplementary figures and images for: Listeria monocytogenes Dampens the DNA Damage Response
Source: PLoS Pathog. 2014 Oct 23;10(10):e1004470. doi: 10.1371/journal.ppat.1004470 (PMC4207825; doi:10.1371/journal.ppat.1004470)

Figure S1

A

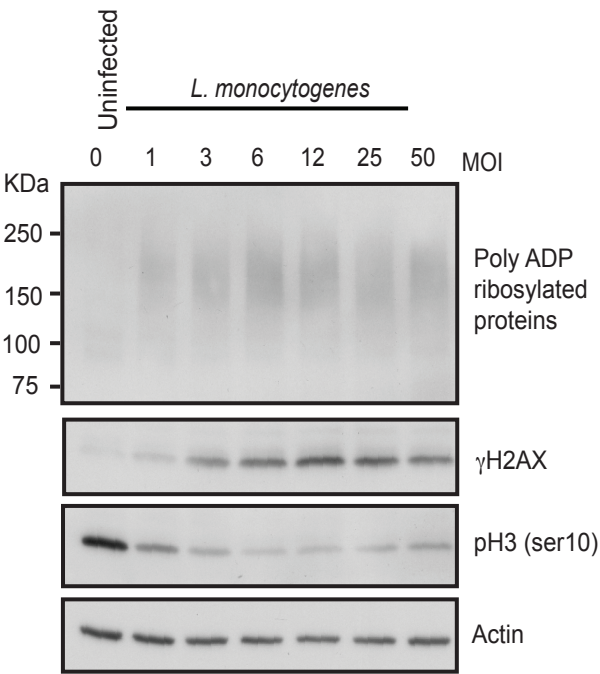

B

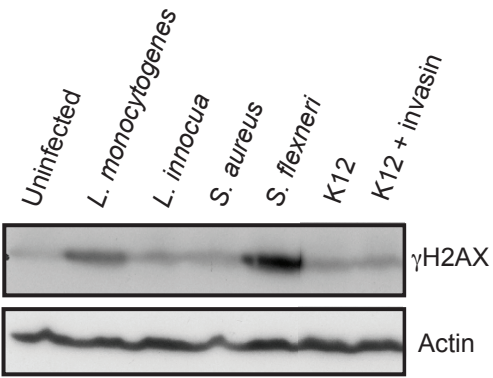

Supplement: Figure S1 — Levels of poly-ADP ribosylated proteins and γH2AX as a function of multiplicity of infection and bacterial strains. (A) HeLa cells are infected with L. monocytogenes using the indicated multiplicity of infection (MOI) and harvested for immunobloting after 24 h of infection. (B) HeLa cells are infected with the indicated bacterial strains and harvested for immunobloting after 24 h of infection. (PDF) [file ppat.1004470.s001.pdf]

Figure S2

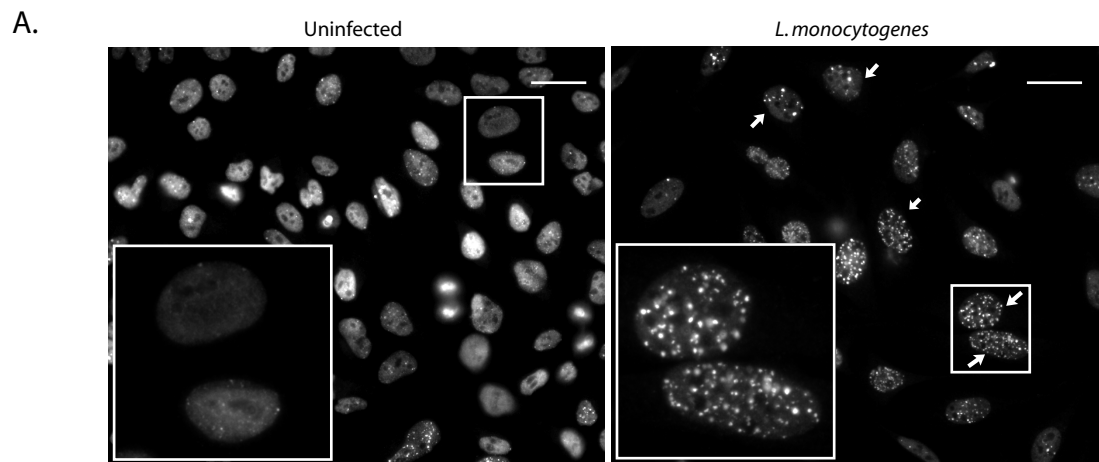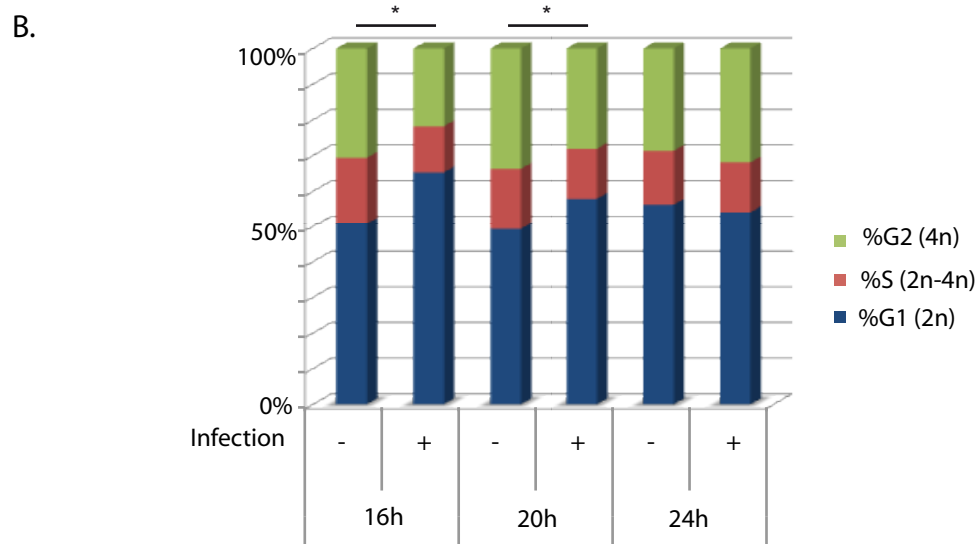

|               | 16h          |                  | 20h          |                  | 24h          |              |
|---------------|--------------|------------------|--------------|------------------|--------------|--------------|
| infection     | -            | +                | -            | +                | -            | +            |
| % G1 (2n)     | 50,8 +/- 5,8 | 65,3 +/- 3,5 (*) | 49,3 +/- 4,3 | 57,9 +/- 3,8 (*) | 55,1 +/- 4,5 | 54,1 +/- 7,2 |
| % S (2n - 4n) | 18,5 +/- 0,4 | 12,9 +/- 0,9 (*) | 17,0 +/- 1,5 | 14,03 +/- 0,8    | 14,8 +/- 2,9 | 14,0 +/- 1,5 |
| % G2 (4n)     | 30,6 +/- 5,9 | 21,8 +/- 2,9     | 33,7 +/- 5,8 | 28,1 +/- 3,1 (*) | 30,1 +/- 4,3 | 31,9 +/- 6,2 |

Supplement: Figure S2 — Effect of infection with L. monocytogenes on 53BP1 foci and the host cell cycle. (A) HeLa cells are infected with L. monocytogenes for 24 h. Immunofluorescence using an antibody specific to 53BP1 is shown. Arrows show cells with more than 3 foci of 53BP1 which are quantified in figure 1. Scale bar is 30 µm, insert is 2.5 times larger than box in original image. (B) Cell cycle quantification of HeLa cells infected (+) or not (−) with L. monocytogenes for the indicated times. In the table the numbers used to draw the graph are shown +/− SEM. (* p<0.05). (PDF) [file ppat.1004470.s002.pdf]

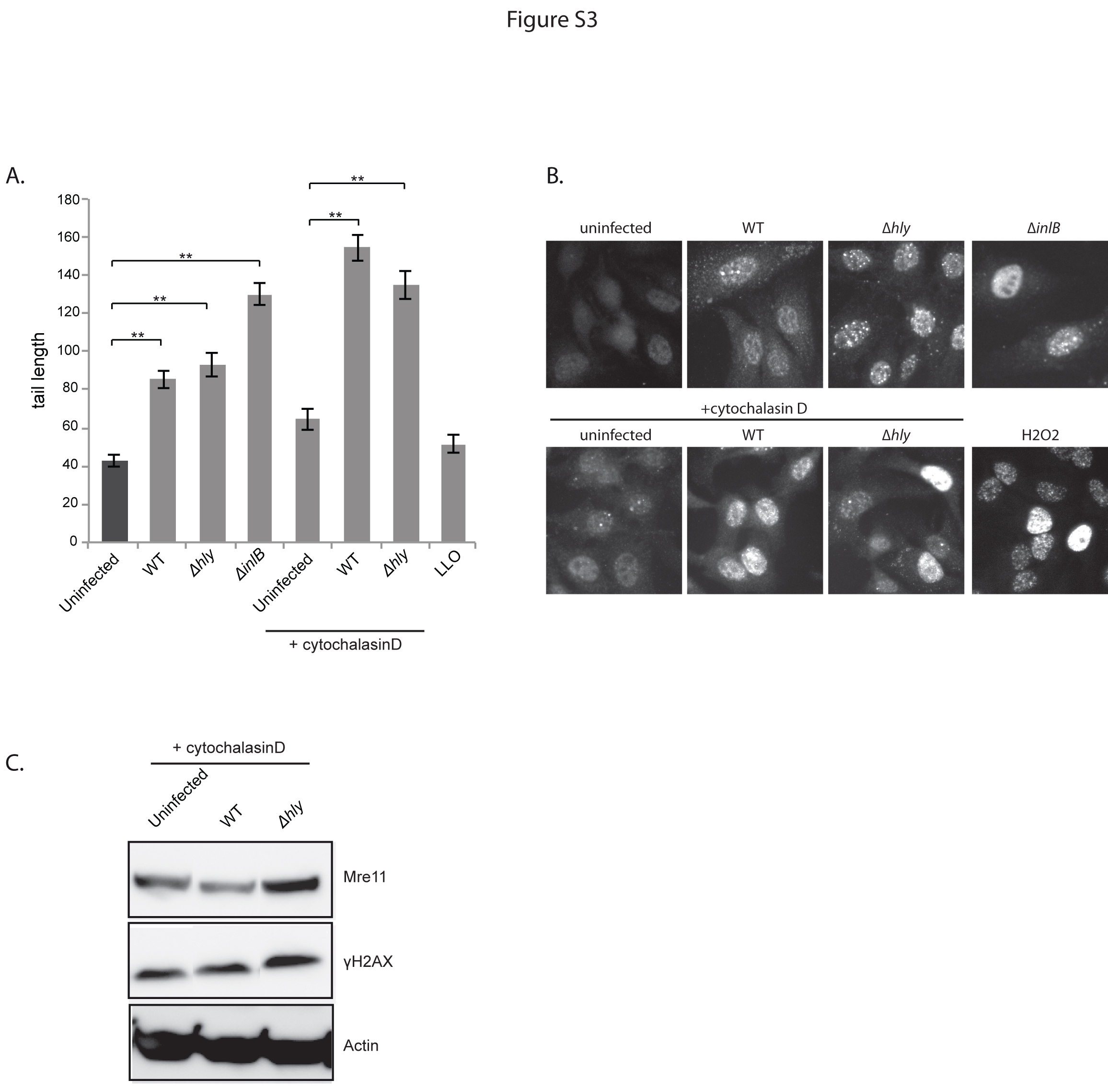

Supplement: Figure S3 — L. monocytogenes mediates its effect on the DDR from the outside of the cell. (A) Quantification of comet assays performed on HeLa cells infected with the indicated strain for 24 h. Cytochalasin D treatment was performed for 15 minutes prior to infection. Each bar in the histogram is an average of at least 30 nuclei from at least 3 independent experiments. Quantifications show the mean +/− SEM (** indicates p<0.01). (B) Immunofluorescence of γH2AX. Each box is 100 µm in length. (C) Immunoblot image representative of 3 independent experiments. (JPG) [file ppat.1004470.s003.jpg]

Figure S4

A.

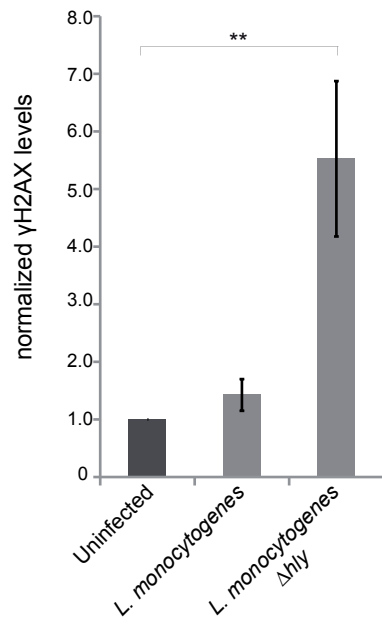

B.

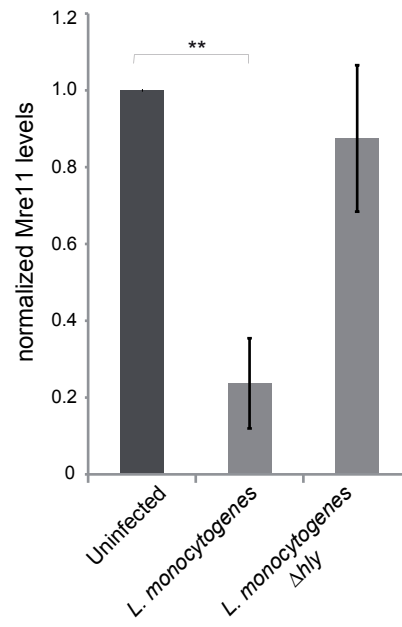

Supplement: Figure S4 — γH2AX and Mre11 levels in JEG3 cells. (A) and (B) JEG3 cells were infected with the indicated strain of Listeria for 24 h. Cell extracts were harvested for immunoblotting. Quantifications are normalized to actin and to the uninfected sample. All quantifications in graphs show the mean +/− SEM (** p<0.01). (PDF) [file ppat.1004470.s004.pdf]

Figure S5

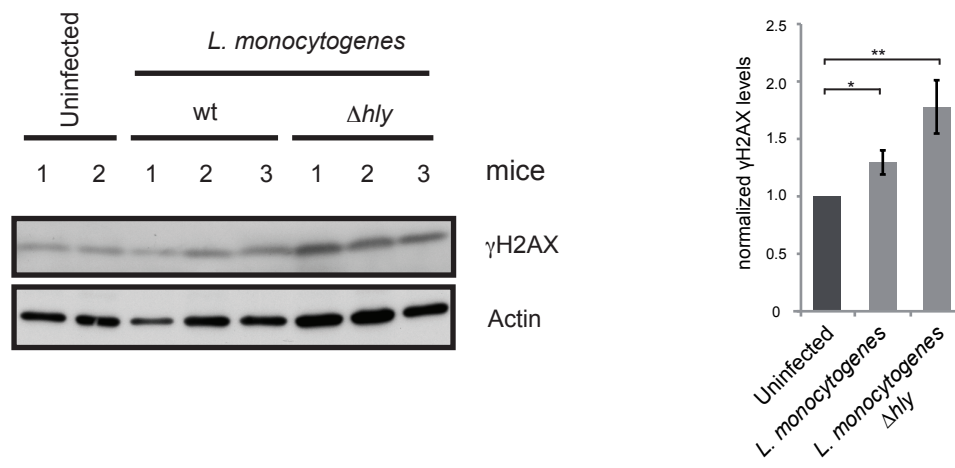

Supplement: Figure S5 — γH2AX in vivo. Immunoblot of peritoneal content from C57Bl/6J mice infected with Listeria for 6 hours. n = 6 mice per condition. Quantifications in graphs show the mean +/− SEM (* p<0.05, ** p<0.01). Recovered colonie forming units for each conditions were as follows: WT, 46+/−29 and Δhly, 45+/−19. (PDF) [file ppat.1004470.s005.pdf]

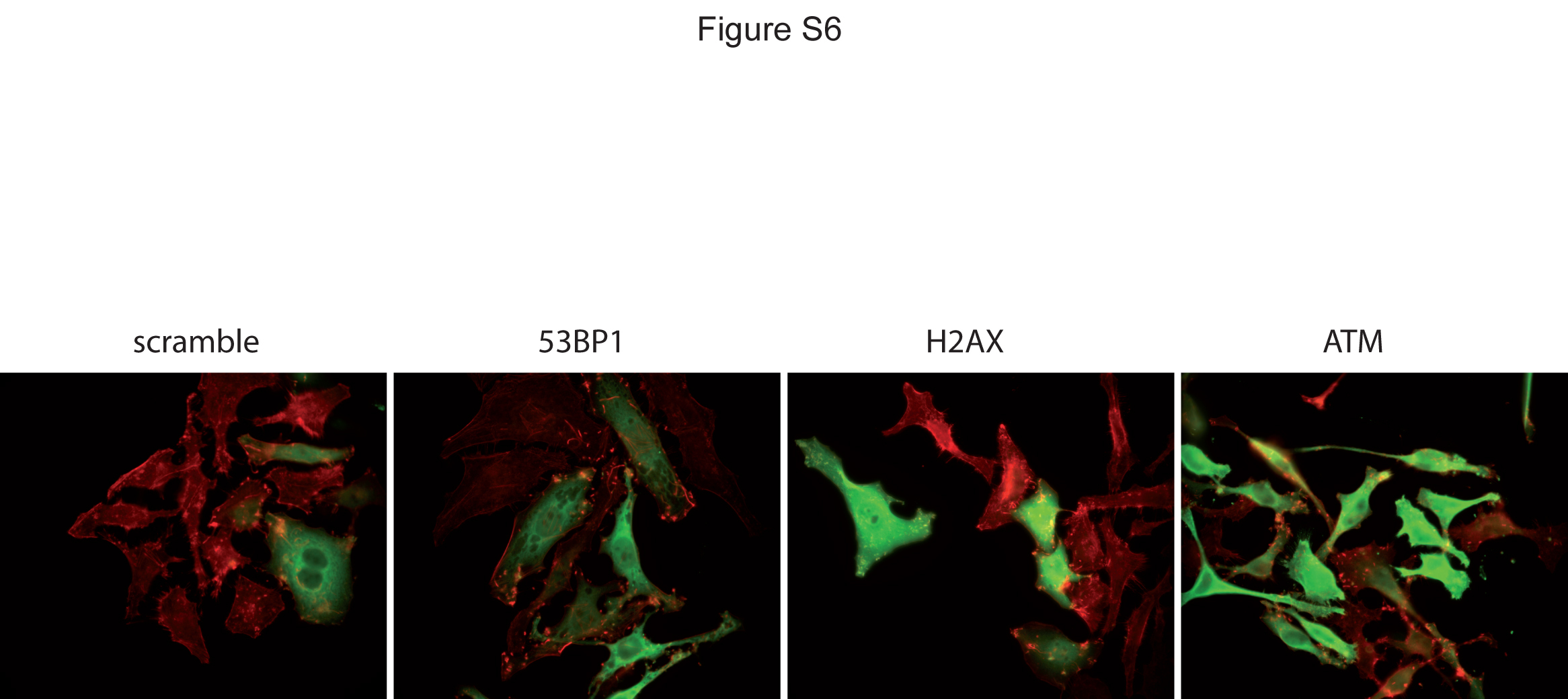

Supplement: Figure S6 — Infection levels in siRNA treated cells. HeLa cells are transfected with siRNA (indicated above each image) and infected with L. monocytogenes for 24 h. Immunofluorescence is performed with anti-InlC antibody (green), which measures the level of infection and phalloidin (red), which stains for actin and shows the cell cytoskeleton as well as bacterial induced actin polymerization. (JPG) [file ppat.1004470.s006.jpg]
